# Role of Complement in the Onset of Age-Related Macular Degeneration

**DOI:** 10.3390/biom13050832

**Published:** 2023-05-13

**Authors:** Niloofar Piri, Henry J. Kaplan

**Affiliations:** 1 Department of Ophthalmology, School of Medicine, Saint Louis University, St. Louis, MO 63104, USA; 2 Departments of Ophthalmology and Biochemistry & Molecular Biology, School of Medicine, Saint Louis University, St. Louis, MO 63104, USA; henry.kaplan@health.slu.edu

**Keywords:** age-related macular degeneration, AMD, complement pathway, drusen, GA, geographic atrophy

## Abstract

Age-related macular degeneration (AMD) is a progressive degenerative disease of the central retina and the leading cause of severe loss of central vision in people over age 50. Patients gradually lose central visual acuity, compromising their ability to read, write, drive, and recognize faces, all of which greatly impact daily life activities. Quality of life is significantly affected in these patients, and there are worse levels of depression as a result. AMD is a complex, multifactorial disease in which age and genetics, as well as environmental factors, all play a role in its development and progression. The mechanism by which these risk factors interact and converge towards AMD are not fully understood, and therefore, drug discovery is challenging, with no successful therapeutic attempt to prevent the development of this disease. In this review, we describe the pathophysiology of AMD and review the role of complement, which is a major risk factor in the development of AMD.

## 1. Introduction

Age-related macular degeneration (AMD) is the leading cause of severe loss of central vision in people over age 50 [1]. It is estimated that nearly 20 million Americans have some form of AMD [2]. Currently, over 200 million people are affected worldwide, and it is estimated that this number will double by 2040 [3,4,5]. In Western countries, nearly 22% of people over 70 and 34% over 80 suffer from AMD in at least one eye [5]. Patients gradually lose central visual acuity, compromising their ability to read, write, drive, and recognize faces, all of which greatly impair their daily life activities [6]. Studies have also shown that quality of life is significantly affected in AMD patients, with less life satisfaction, more stress, less physical activity, and worse depression levels [7,8].

### 1.1. Anatomy of AMD

**Figure 1:**
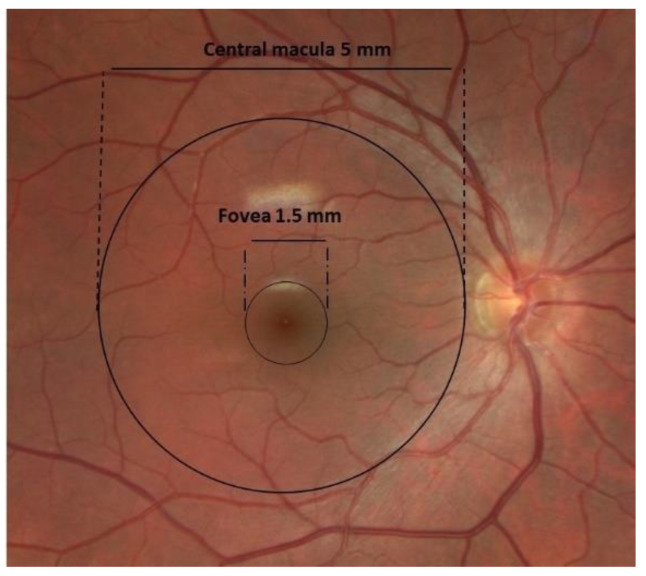
Clinical fundus photograph of the macula and fovea and their dimensions.

The pathology of AMD is restricted to the outer retinal layers and the retinal pigment epithelium (RPE) of the macula [9]. The central macula, or central posterior retina, measures 5 mm in diameter, with the highest density of cone photoreceptors located in the fovea, a 1500-micron area responsible for central visual acuity and color vision [10] (Figure 1). The fovea has a unique anatomical structure, both because of the high density of cone photoreceptors and the absence of retinal blood vessels [11]. The cells in the fovea receive nutrition from the underlying choroidal circulation and the adjacent perifoveal capillary network. The absence of an inner retinal vasculature allows improved light absorption by the cone photoreceptor cells, providing high-resolution vision [12].

RPE cells are a polarized monolayer of cuboidal cells between the neurosensory retina and Bruch’s membrane (BM). They are of vital importance for the homeostasis of the entire retina, but especially the fovea. Nutrients and oxygen are supplied to the macula by the choroidal circulation through Bruch’s membrane and the RPE. Metabolic waste products of the retina are also transported through the RPE/BM complex into the choroidal circulation [11]. RPE cells are also important in the phagocytosis of shed photoreceptor outer segments, which is an essential part of the visual cycle [13] and have an important role in protecting the retina from oxidative stress due to large numbers of melanosomes that absorb light [14]. RPE is separated from the underlying choroid by Bruch’s membrane (BM), which is composed of five layers—the basement membrane of RPE cells, the inner collagenous layer, the middle elastic layer, the outer collagenous layer, and the basement membrane of the choriocapillaris endothelial cells [15]. In addition to being a structural support for the RPE, BM also contributes to the outer blood–retinal barrier and the selective transport of molecules and proteins. Therefore, any changes in the choroid–BM–RPE complex will affect the natural homeostasis of photoreceptors. These changes may contribute to the development of AMD [12].

### 1.2. Pathophysiology of AMD

Major changes in BM contribute to macular damage, including increased thickness, which is a result of increased deposition and the cross-linking of collagen fibers, and decreased permeability, resulting in decreased RPE metabolic function [12,16,17]. It has also been observed that with aging, there is a deposition of advanced glycated end products (AGEs), i.e., glycated or oxidized lipoproteins [18], which can trigger inflammation, as well as the presence of leukocytes in the choroid underneath the macula in AMD.

RPE cells lose melanosomes, and mitochondria become more prone to oxidative stress with aging [19,20]. This is associated with major changes in the choroid. The normal choroid has high blood flow and can change rapidly, depending on the physiologic demand of the RPE and neurosensory retina. Aging leads to decreased blood flow, as well as the thinning of the choroid [21]. Therefore, flexibility decreases, and the choroid cannot rapidly respond to changes in blood flow required for the physiologic demand. Decreased blood flow results in a decreased support of oxygen and nutrients to the retina. The resultant hypoxia and lack of sufficient nutrient support drives RPE cells to extreme metabolic stress. Additionally, reduced blood flow impairs the removal of photoreceptor and RPE waste products, which are now deposited in the BM, leading to drusen formation [22].

“Drusen” are extracellular deposits between the basal lamina of the RPE and the inner collagenous layer of BM [23,24]. They consist of many different components, and the composition varies with the disease stage. More than 40% of its contents include neutral lipids and esterified and unesterified cholesterol [22]. In addition, more than 129 proteins have been identified in drusen [25], including inflammatory and complement factors, such as vitronectin, serum beta amyloid protein, tissue inhibitor of metalloproteinase 3 (TIMP3), apolipoproteins (E, B, A-I, C-I, and C-II), immunoglobulin light chains, complement proteins (C5 and the C5b-9 complex), proteins involved in complement regulation, and zinc and iron ions [22,25,26].

### 1.3. Clinical Presentation

**Figure 2:**
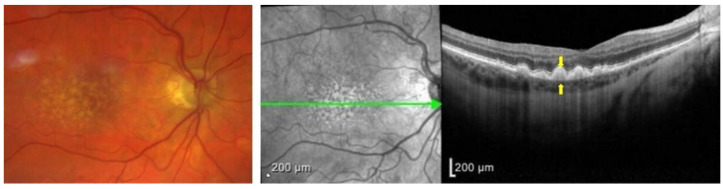
(**Left**) Color fundus photograph of a patient with intermediate AMD demonstrating soft drusen, with indistinct borders. (**Right**) Optical coherence tomography (OCT) imaging of the central macula in the same patient demonstrating an optical section of all retinal layers. Notice the hyperreflective deposits between the RPE (top arrow)and the BM (bottom arrow), which represent drusen from the left panel.

Upon clinical examination of the fundus, drusen present as small, yellowish-white spots underneath the retina in the central macula. They are classified clinically in the Wisconsin grading system as hard or soft drusen. Hard drusen are defined as well-defined yellow spots that are 1–63 microns in diameter. Soft drusen are larger than 125 microns, or if they are 63–125 microns, they have visible thickness. Soft drusen can have either distinct or indistinct borders (Figure 2) [27]. They are linked with a higher risk of choroidal neovascularization (CNV) because they include C3a and C5a and can trigger the overexpression of the vascular endothelial growth factor (VEGF) in RPE [28].

Hard drusen are often present in small numbers with aging but increase in AMD patients. This implies that other mechanisms underlie AMD pathogenesis beyond the simple presence of drusen themselves [12]. As the disease progresses from the early to the intermediate stage of AMD, the number and size of drusen increase, and RPE changes occur. With further progression, two late sight-threatening stages are recognized: geographic atrophy (GA, or end-stage dry AMD) and neovascular AMD (nAMD or wet AMD). GA is characterized by the death of RPE cells, the overlying photoreceptors, and the underlying choriocapillaris, whereas nAMD is most frequently characterized by the growth of abnormal choroidal blood vessels into the sub-RPE or subretinal space (CNV) [12,29]. GA clinically starts as horseshoe patches of RPE atrophy in the perifoveal area and gradually enlarges to involve the fovea. It starts in an area with drusen, and as the disease progresses, regression of drusen occurs [30,31].

### 1.4. Risk Factors for AMD

The pathogenesis of AMD is multifactorial, involving a combination of genetic and environmental factors in its development. The most important modifiable environmental factor is cigarette smoking, which contributes significantly to the development, as well as the progression, of the disease and oxidative damage [32]. Smoking increases the risk of AMD 2–4-fold. The risk is dose dependent, and after cessation of smoking, the risk of AMD decreases. It has been demonstrated that 20 years after quitting smoking, the risk is minimal [33]. Other environmental risk factors include cataract surgery, diet, cardiovascular disease, hypertension, and body mass index (BMI). The Mediterranean diet has been shown to decrease the risk of AMD, presumably because of the high content of antioxidants in vegetables and fruits, which are integral components of this diet [12,34,35,36]. It has also been observed that eating fish protects against AMD [37]. The Age-Related Eye Disease Study (AREDS), a large, multicenter clinical trial, demonstrated the most convincing evidence for a connection between oxidative stress and AMD [38,39]. They showed that certain vitamins and antioxidants decreased the rate of progression of intermediate-stage AMD by 25% in an average follow-up of 6.3 years.

Studies into the molecular components of drusen already hinted that AMD might have an immune component before any particular gene or biological pathway was definitively connected to the condition. This hypothesis was proposed after proteins related to inflammation and/or other immune-related molecules, such as complement system components, were discovered in drusen [40].

Twin and family studies provided evidence that AMD has a significant hereditary component. Twin studies found that monozygotic pairs had a high concordance of AMD, even double that of dizygotic pairs, and they predicted that the heredity component of AMD could be as high as 45 to 70% [40,41,42,43].

Genetic associations with AMD are well documented in different genome-wide association analyses (GWAS). The strongest genetic contributors to AMD with the highest odds ratios are variants associated with complement factor H (CFH)—complement factor H-related (CFHR) 5 on chromosome 1q32 (Chr1 locus), and age-related maculopathy susceptibility 2 (ARMS2) and high-temperature requirement factor A1 (HTRA1), two tightly-linked genes located on chromosome 10q26 (Chr10 locus) [44,45,46,47]. The most well-known genetic risk factor for AMD is the complement factor H Y402H (Tyr402His) variation. While another missense mutation of CFH, I62V (Ile62Val), is more common in Asian populations, this mutation is specifically connected to AMD susceptibility in Caucasians [48,49,50]. The primary function of the glycoprotein CFH, which has 20 domains called short consensus repeats (SCR), is to prevent the activation of the alternative complement pathway [51].

Complement FH, a significant complement system inhibitor, not only competes with CFB for binding to C3b, speeding up the dissociation of the resultant alternative system C3 convertase (C3bBb), it also functions as a cofactor to facilitate the factor I (FI)-mediated inactivation of C3b [52,53].

In addition to CFH, several complement genes are involved, including CFB, CFI, C3, and C2 [54]. Since the latter discoveries, a genetic association between AMD and the complement system was further strengthened. At least 34 genomic loci are also associated with AMD pathogenesis, including genes related to cholesterol metabolism, extracellular matrix remodeling, and collagen structure [55].

Many of these genes are involved in either enhancing activation or decreasing deactivation of the active complement factors, therefore resulting in the dysregulation of complement control, with ultimate damage to the host tissue.

These genetic association studies led to the identification of a dysregulated alternative complement pathway as a main driver of AMD, and more recently, they served as useful tools for proposing associated candidate therapeutic targets [12]. Immunohistochemical studies supported this conclusion [56,57]. Clearly, inflammation plays a role in AMD pathogenesis and is referred to by some investigators as an example of para-inflammation [11,54].

In this study, we outlined potential scenarios for how AMD might develop and worsen as a result of complement system malfunction.

## 2. AMD and the Complement System

**Figure 3:**
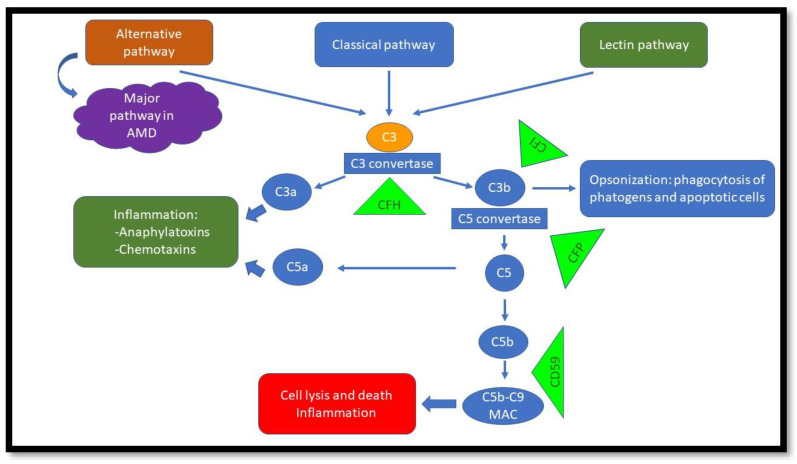
Complement pathways. The alternative pathway is important in AMD. Green triangles demonstrate regulators/inhibitors of the specific pathways. Malfunction will lead to overactivation of complement and, ultimately, cell damage.

The complement system is a crucial component of the innate immune response that protects the host against invading pathogens, such as bacteria. It has three pathways: classic (antigen–antibody complex), lectin-dependent (mannose polysaccharides on microorganisms), and alternative (pathogen cell surfaces) [58]. Each pathway has its own distinctive factors, but they all converge and ultimately lead to the cleavage of complement factor 3 (C3) to C3a and C3b. C3a is anaphylatoxin and induces inflammation, and C3b opsonizes cells and labels them for phagocytosis. The complement cascade continues and ultimately forms the membrane attack complex (MAC), which includes (C5b-C9). The MAC penetrates the cell membrane and results in cell death [58,59,60]. The formation of the MAC can be inhibited by complement inhibitors, such as CD59 [61]. Under normal conditions, host cell regulatory proteins deactivate active complement components and prevent damage to host cells [59](Figure 3).

The complement system can be considered a double-edged sword. On one side, under normal conditions, it protects the body against foreign pathogens and modifies the surveillance of immune responses; on the other side, under abnormal conditions, its dysregulation or dysfunction can lead to uncontrolled inflammatory responses and can damage host tissue, resulting in disease [62].

The alternative pathway is activated in two different ways. The first one is called “tickover”, which is the spontaneous hydrolysis of C3 into C3 (H2O) in the fluid phase. The second one is by C4b2a (C3 convertase), which results in the cleavage of C3 to C3a and C3b in the solid phase [63]. The alternative pathway is responsible for the majority (80–90%) of the terminal pathway activation [64]; therefore, overactivation of this pathway may have a major role in disease pathogenesis. Early investigations initially debated whether systemic or local complement activation caused disease in the eye. However, it may well be that complement-mediated molecular processes causing AMD are a combination of systemic complement proteins, as well as locally synthesized complement proteins [12]. Experimental studies in animal models suggested the idea above.

The most commonly used animal model mimics wet AMD and is induced by retinal laser photocoagulation in rodents. Laser-induced CNV can be suppressed by blocking complement activation via local or systemic mechanisms. Inhibiting C3a, C5a, CFB, and MAC or administering the complement regulating molecules CD59 and CFH can prevent the formation of CNV in animal models [65,66,67]. In CFH-deficient mice, excessive alternative pathway activation resulted in the enlargement of the induced CNV [68]. It was also shown that mice lacking the alternative complement pathway (CFB-deficient) had decreased rates of CNV, compared to animals lacking other complement pathway constituents [67,69].

In individuals with AMD, several complement system activation products have been identified to be increased systemically [70]. Patients with intermediate or late dry AMD (i.e., central GA or inactive CNV) have higher levels of systemic complement activation, compared to both controls and early AMD [12]. In contrast, the level of systemic complement activation in wet AMD is relatively low [12,70]. Study of donor eyes from patients with the CFH gene showed increased deposits of local C3b before clinical manifestations of the disease. Thus, poorly controlled complement turnover may start earlier than clinical disease manifestation within the retina [70].

Five AMD risk alleles have been recognized involving the alternative complement pathway, implying a role for innate immunity in disease development. Additionally, the pathogenesis of early AMD may be different than that of late-stage AMD [35]. A recent GWAS identified a gene variant near CD46 almost exclusively associated with early AMD [71].

Interestingly, complement products and the membrane attack complex (MAC) have been found in the choroids of eyes from young donors, as well as those without genetic risks for AMD. This suggests that low-grade complement activation is likely required for the normal homeostasis of the retina/RPE complex [12,72,73,74], similar to observations made in the anterior chamber of the eye [75]. The complement system may assist in the removal of waste products, such as the shed photoreceptor outer segments. MAC levels increase with aging, possibly leading to bystander complement-mediated injury of the tissue [12,72,73,74].

A frequent consequence of complement activation is the recruitment and activation of immune cells and the release of the anaphylatoxins C3a and C5a [12]. The immune cells involved in AMD comprise resident microglial cells, as well as circulating lymphocytes, monocytes/macrophages, and mast cells. Stimulation of monocytes by C3a leads to the secretion of multiple cytokines, possibly leading to tissue damage. Mast cell degranulation by C3a and C5a can lead to the release of proteases, tryptase, and chymase, with resultant damage to the extracellular matrix (ECM) [76].

Lipid deposition in the retina has also been linked to both systemic and local complement systems dysregulation [77]. Studies on the serum of AMD patients demonstrated a substantial correlation between elevated large and extra-large high-density lipoprotein (HDL) levels and decreased, very low-density lipoprotein (VLDL) and amino acid levels. These changes were associated with elevated serum complement activation [78].

CFH has been shown to be present in large HDL particles but not in small or medium HDL molecules [79]. Whether this is pro-inflammatory or suppressive for inflammatory responses is not yet clear. Entrapment by HDL might decrease the availability of circulating CFH to deactivate the complement cascade [79]. On the other hand, the presence of CFH in drusen might suppress inflammation locally in the retina. The latter has supportive evidence, since it has been shown that factor H binds the native low-density lipoprotein (LDL) and the oxidized LDL; this binding affinity is reduced in patients with the CFH high-risk 402H variant. As a result, increased levels of oxidized LDL may lead to the upregulation of inflammatory cytokines and the start of an inflammatory cycle that results in tissue damage [80].

It was also observed that disturbed RPE metabolism is important in the pathogenesis of AMD. Compared to RPE cells from healthy controls, primary RPE cells isolated from AMD patients were shown to have severely impaired energy metabolism [81].

Complement dysregulation associated with the CFH variant is associated with reduced energy metabolism in RPE cells [82]. Complement dysregulation was associated with mitochondrial damage in RPE cells [83]. Compared to RPE cells from donors with the low-risk 402Y genetic variation, RPE cells from AMD donors harboring the high-risk 402H variant exhibited greater mtDNA damage [83]. In the CFH (cfh-/-) knock-out mouse model, abnormally large mitochondria were shown in the RPE and photoreceptors. At the same time, the amount of mitochondrial DNA (mt DNA) decreased, which is a sign of energy dysregulation confirmed by lower adenosine tri-phosphate (ATP) production [82]. Regarding an oxidative stress response, there seems to be a reciprocal interaction between the complement system control and energy metabolism [12]. In a cytoplasmic hybrid (cybrid) model, with mitochondria from AMD patients and ARPE19 devoid of mitochondria, changes in complement components were demonstrated. Complement activators, including CFB, were found to be increased, and complement inhibitors, including CFH, were found to be decreased [84].

Another crucial component of RPE homeostasis is the lysosome–autophagy machine, which is involved in recycling metabolites and degrading damaged organelles, including shed photoreceptor outer segments [85,86]. If the lysosome–autophagy machine in RPE is affected, resultant waste products within the cell will accumulate, rather than being recycled/cleared, with the possible onset of AMD [86]. Figure 3 is a simplified diagram of complement pathways and the major regulators involved in AMD.

## 3. Therapeutic Strategies

There is no effective treatment to prevent the development of either type of AMD, and although intravitreal injection of anti-VEGF medications can stabilize and improve vision in the majority of patients with wet AMD, the treatment is costly, it requires monthly treatments for several years, and up to 58% of Medicare patients discontinue treatment within the first year [87]. Additionally, anti-VEGF treatments remain ineffective for some patients, and a significant proportion of these patients still develop severe visual loss and progress to legal blindness over time [88].

On 17th February 2023, the FDA approved the first intravitreal injection therapy for geographic atrophy. Pegcetacoplan is a drug that targets C3, and the 2-year Oak and Derby studies leading to FDA approval demonstrated efficacy, compared to sham injections and efficacy, over time, with the greatest benefit resulting in a 36% growth reduction in the GA lesion from 18–24 months in the Derby study [SYFOVRE (pegcetacoplan injection) [package insert]. Waltham, MA: Apellis Pharmaceuticals, Inc.; 2023]

Given the strong genetic and biological evidence that dysregulation of the complement system is a major driver of AMD pathogenesis, it is not surprising that there is increasing interest in complement therapeutic targets to prevent progression of the disease [12].

Early clinical trials with intravenous administration of eculizumab, which targets C5 and is FDA approved for use in paroxysmal nocturnal hemoglobinuria (PNH) and atypical hemolytic uremic syndrome (HUS), failed to show any effects in GA patients during a phase II clinical trial [89].

Several factors may have possibly prevented successful eculizumab treatment of GA, including the lack of stratification of patients based on their genetic risk, intervening too late in the disease process, or low drug dosage at the site of disease because of systemic administration rather than at the extracellular matrix of the choriocapillaris. Certainly, local administration of drugs directly into the eye holds multiple possible benefits and is now the preferred route of delivery in several current clinical trials for retinal disorders [12].

On the other hand, local administration is associated with several caveats. Introducing drugs to an immune-privileged site, such as the eye, with the inhibition of complement components may increase the risk of endophthalmitis, particularly with the requirement for frequent, regular intravitreal injections [12,90].

More than 14 complement inhibitors have been developed so far that target major components of the complement system (C3, C5) or regulators of the complement system (CFD, CFI). More than 40 clinical trials are either completed or on-going in the treatment of dry AMD with complement inhibitors [62]. Most of these trials are using the enlargement of the GA lesion or loss of visual function as therapeutic endpoints.

APL-2 and Zimura, respectively, target C3 and C5 and are the two most advanced treatments, since both are in phase III trials. Both drugs are administered locally via intravitreal injection. They stop the formation of C5a and MAC and, ultimately, cell lysis. APL2 also prevents the formation of C3a, since it works more upstream. In phase II trials, both APL-2 and Zimura were associated with decreased GA growth rates—29 and 27.8%, respectively. At the same time, both showed a small increased incidence of choroidal neovascularization in the treated eyes, compared with the sham [91,92]. The latter may be related to polyethylene glycol (PEG) in the formulation of both drugs. PEG has been used in the past to induce CNV in mice [93]. As mentioned earlier, on 17th Feb 2023, the FDA approved the first intravitreal drug for the treatment of geographic AMD, whether involving the fovea or not, after the evaluation of the two-year results. Pegcetcoplan/APL-2 works by targeting C3. The drug will be available shortly to slow down the progression and growth of the GA lesion, but there is no reversal effect.

Interestingly, none of the current studies address the downstream effects related to the modulation of the complement pathway, such as the effect on the immune response, RPE cell metabolism, and RPE attachment to the ECM and BM [12]. In the future, a combined mechanism that addresses both upstream and downstream genes in target cells might be a more effective way to treat and/or prevent the development of AMD.

## 4. Conclusions

In conclusion, age-related macular degeneration is a complex disease that leads to central vision loss. Genetic and environmental factors are involved in the development of the disease. The mechanisms by which these factors interact to develop the disease are not fully understood; however, it is presumed that the complement system has a major role in the onset and development of the disease. Focusing future studies on the complement system in the retina and the RPE may further elucidate molecular changes at a cellular level that lead to disease development.

## Data Availability

Not applicable.
